# Supplementary material for: Convergence of cMyc and β‐catenin on Tcf7l1 enables endoderm specification
Source: EMBO J. 2015 Dec 16;35(3):356–68. doi: 10.15252/embj.201592116 (PMC4741304; doi:10.15252/embj.201592116)
Supplement: Supplementary file 1 — Expanded View Figures PDF [file EMBJ-35-356-s001.pdf]

Expanded View Figures

Figure EV1. GSK3 inhibition promotes the production of definitive endoderm.

A Schematic representation of the two-step endoderm differentiation protocol for naïve ES cells. Basal media N2B27 and novel basal media SF5 are shown with added cytokines and small-molecule inhibitors. Further details are provided in Materials and Methods.

B Gene expression profiling by qRT–PCR at days 1–4 of differentiation. Average and SD of three independent experiments are shown.

C Assay of *FoxA2* and *Sox17* mRNA by RT–PCR at day 5 of differentiation following pulses of 3 μM CH treatment at days 0–2 (d0-2), days 1–3 (d1-3) and days 0–5 (d0-5) as indicated. Average of three independent experiments are shown.

D Assay of *Sox7* mRNA by RT–PCR at day 7 of differentiation plus or minus 3 μM CH (left panel). Analysis of alpha foetal protein (AFP) by qRT–PCR at day 4 of differentiation plus CH and following an additional 4 days in hepatocyte differentiation conditions (hep diff) (right panel). Flow cytometry quantification of PDGFRa^{+} cells at day 7 of differentiation plus or minus 3 μM CH (lower panel). Average and SD of three independent experiments.

E Flow cytometry quantification of CXCR4^{+} cells at day 7 of differentiation time course plus or minus 3 μM CH and five independent small-molecule inhibitors of GSK3. Average and SD of three independent experiments.

F Flow cytometry quantification of CXCR4^{+} cells at day 7 of differentiation time course of ES cells in control conditions (3 μM CH, 20 ng/ml Activin A, 10 ng/ml FGF4, 1 mg/ml heparin and 100 nM PI103) or minus Activin, minus FGF4/heparin, minus PI103. Percentage of CXCR4^{+} cells is displayed top right. HexRedStar cell was used for this analysis and Hex (red fluorescence) is displayed on the vertical axis.

G Immunostaining for Sox17 in three independent mouse ES cell lines at day 4 to 7 of differentiation in the presence of 3 μM CH. E14: E14Tg2a, HRS: HexRedStar, NOD: derived from non-obese diabetic mice. Scale bars, 200 μm.

H Assay of *Pdx1* and *Ngn3* mRNA by RT–PCR at day 7, 9 and 12 of differentiation. Average and SD of two independent experiments.

I Pdx1 (left panel) and Ngn3 (middle panel) immunostaining at day 9 and day 12 of differentiation, respectively. AFP immunostaining (right panel) at day 12 of differentiation. Scale bars are 200 μm, apart from AFP (100 μm).

© 2015 The Authors

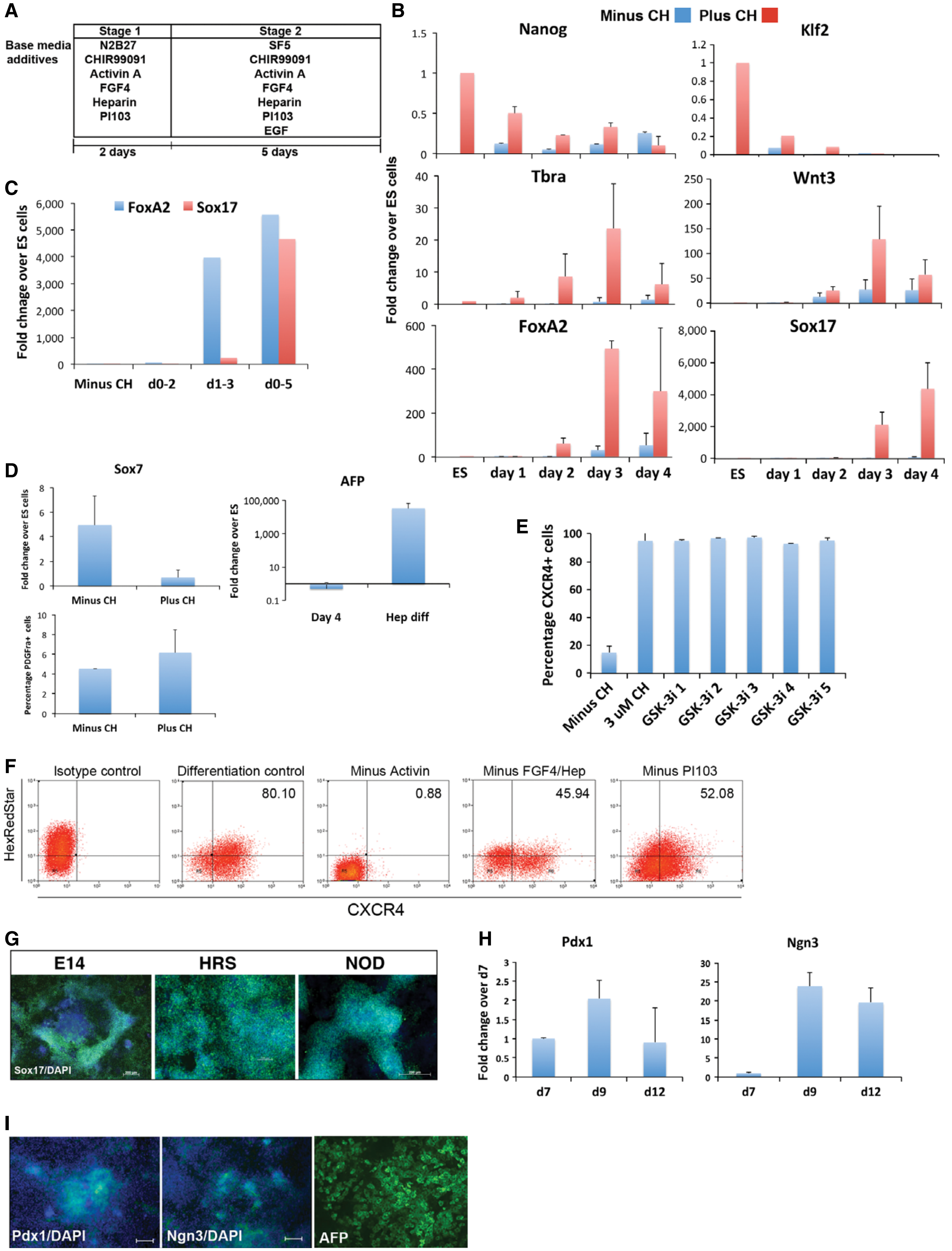
A
Stage 1
Stage 2
Base media
additives
N2B27
CHIR99091
Activin A
FGF4
Heparin
PI103
SF5
CHIR99091
Activin A
FGF4
Heparin
PI103
EGF
2 days
5 days
B
Minus CH
Plus CH
Nanog
Klf2
Tbra
Wnt3
FoxA2
Sox17
Fold change over ES cells
ES
day 1
day 2
day 3
day 4
C
FoxA2
Sox17
Fold chnage over ES cells
Minus CH
d0-2
d1-3
d0-5
D
Sox7
Fold change over ES cells
Minus CH
Plus CH
AFP
Fold change over ES
Day 4
Hep diff
Percentage PDGFra+ cells
E
Percentage CXCR4+ cells
Minus CH
3 uM CH
GSK-3i 1
GSK-3i 2
GSK-3i 3
GSK-3i 4
GSK-3i 5
F
Isotype control
Differentiation control
Minus Activin
Minus FGF4/Hep
Minus PI103
80.10
0.88
45.94
52.08
HexRedStar
CXCR4
G
E14
HRS
NOD
Sox17/DAPI
H
Pdx1
Ngn3
Fold change over d7
d7
d9
d12
I
Pdx1/DAPI
Ngn3/DAPI
AFP

Figure EV1.

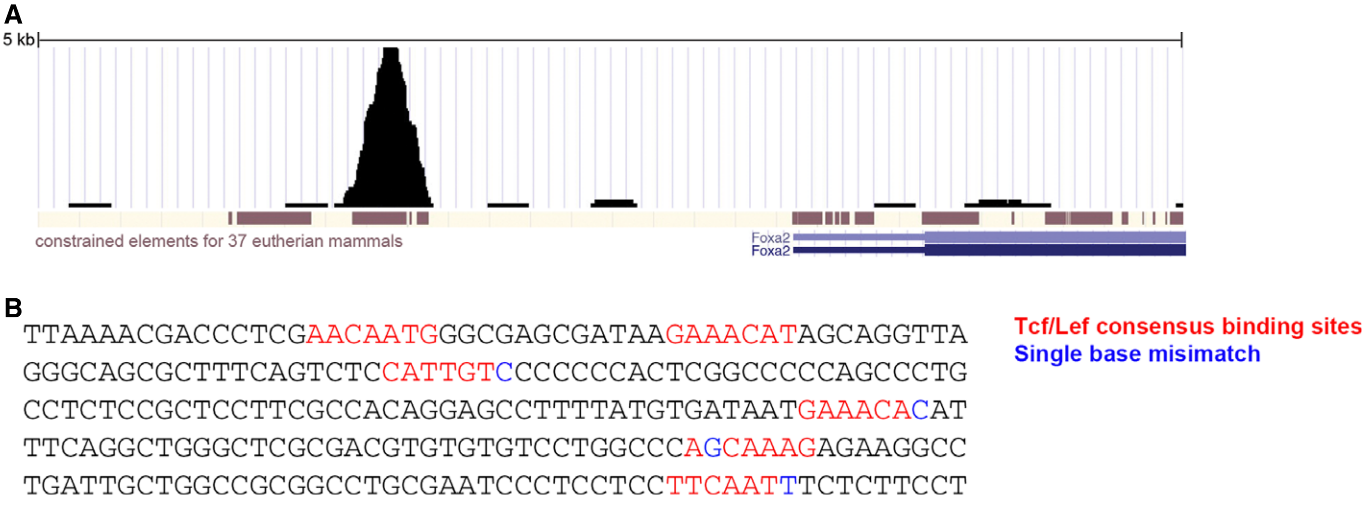

Figure EV2. *FoxA2* genomic region shows evidence of Tcf7l1 binding.
A Genomic sequence proximal to *FoxA2*. Tcf7l1 binding peak as in Fig 4A with regions conserved within eutherian mammals shown in pink boxes below (Flicek *et al*, 2013).
B 250-bp sequence of the Tcf7l1-binding region proximal to *FoxA2* showing 2 consensus Tcf/Lef-binding sites and four other highly similar sites.

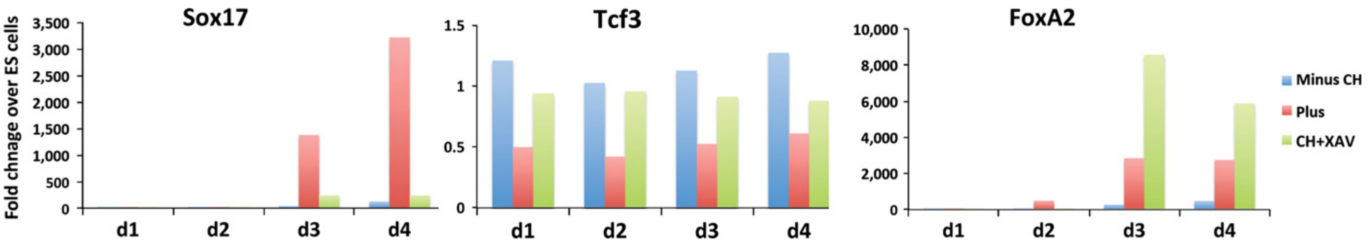

Figure EV3. Blocking Wnt/β-catenin signaling does not inhibit *FoxA2* expression.
Assay of *Sox17*, *Tcf7l1* (Tcf3) and *FoxA2* mRNA by RT–PCR for the first 4 days of differentiation in the presence or absence of 3 μM CH and 3 μM CH plus 1 μM XAV-939.

 © 2015 The Authors

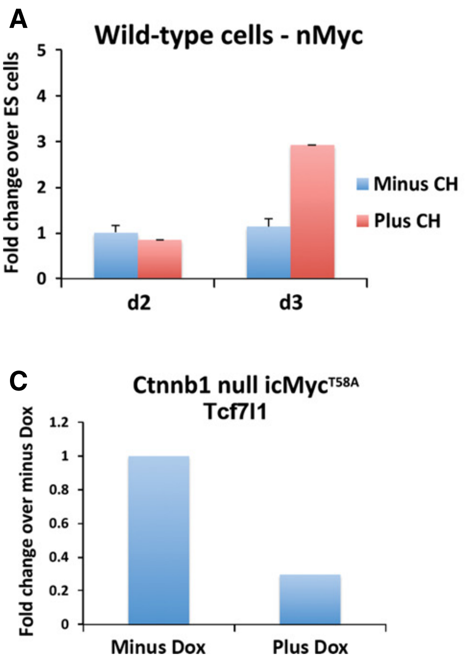

Figure EV4. Repression of *Tcf7l1* by cMyc is independent of β-catenin.

A Assay of *nMyc* mRNA by RT–PCR at day 2 and 3 of differentiation in the presence or absence of 3 μM CH. Average and SD of three independent experiments shown.

B Assay of *cMyc* and *Tcf7l1* mRNA by RT–PCR in cMyc^{T58A}-inducible *Ctnnb1* null cell lines 24 h after DOX treatment. The dose of DOX is indicated in ng/ml.

C Assay of *Tcf7l1* mRNA by RT–PCR in cMyc^{T58A}-inducible *Ctnnb1* null cell line at day 3 of differentiation in the absence of CH following cMyc induction.

© 2015 The Authors

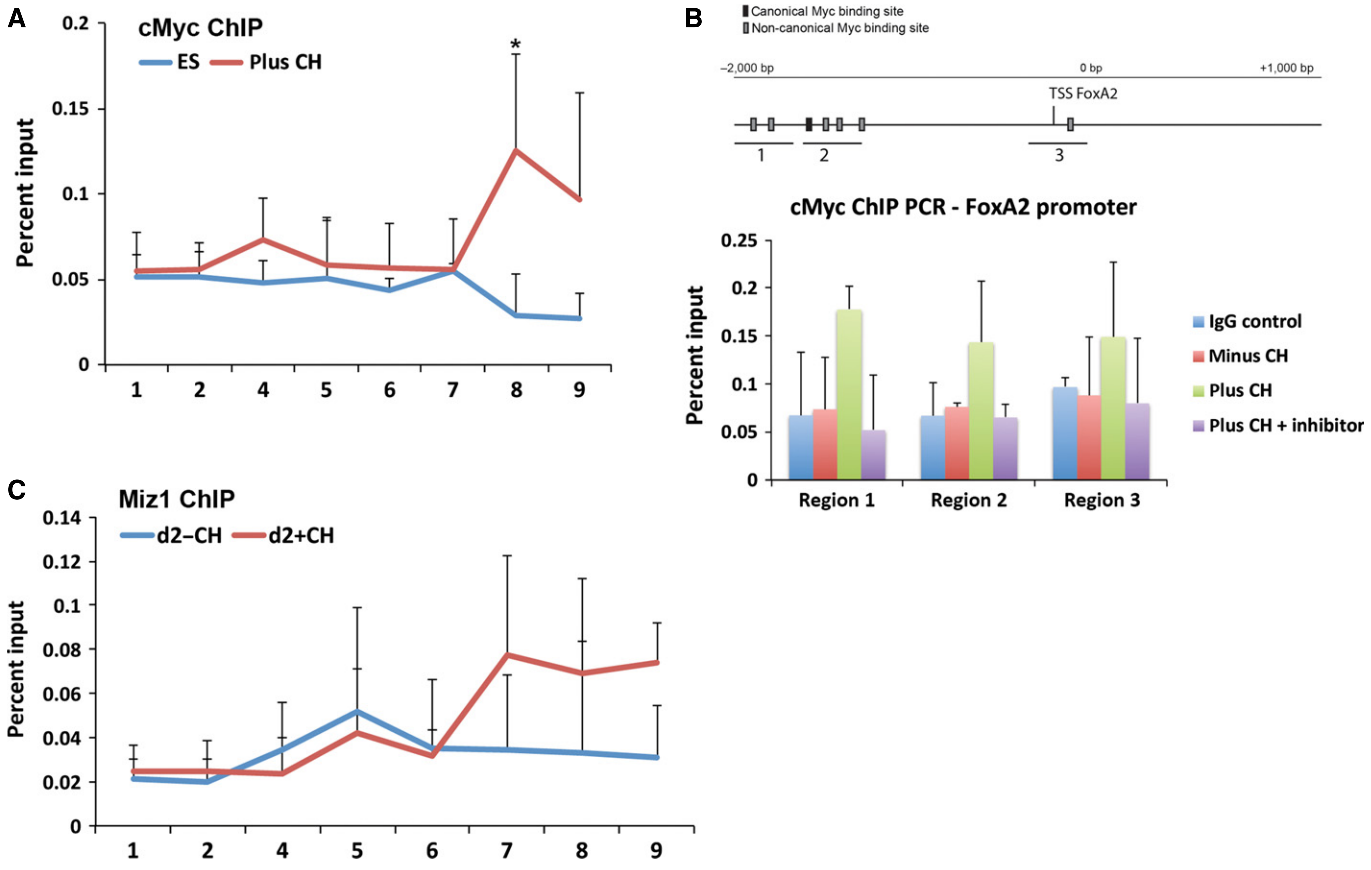

Figure EV5. *FoxA2* is not a direct target of cMyc.

A ChIP for cMyc performed in undifferentiated wild-type ES cells and wild-type cells differentiated for 2 days in the presence of 3 μM CH (plus CH). qPCR was carried out for the regions indicated in Fig 7A. Average and SD of three independent experiments, *$P < 0.05$.

B Schematic representation of the genomic region next to the *FoxA2* transcriptional start site (TSS) (top panel). ChIP for cMyc performed in wild-type ES cells differentiated for 2 days in the absence or presence of 3 μM CH or 3 μM CH plus Myc inhibitor 10058-F4. qPCR was carried out for the regions indicated in the panel above. IgG controls are also shown. Average and SD of three independent experiments (lower panel). Differences between minus CH and plus CH samples did not reach statistical significance.

C ChIP for Mix1 performed in wild-type cells differentiated for 2 days in the absence or presence of 3 μM CH. qPCR was carried out for the regions indicated in Fig 7A. Average and SD for three independent experiments.

 © 2015 The Authors
