# Supplementary material for: Convergence of cMyc and β‐catenin on Tcf7l1 enables endoderm specification
Source: EMBO J. 2015 Dec 16;35(3):356–68. doi: 10.15252/embj.201592116 (PMC4741304; doi:10.15252/embj.201592116)
Supplement: Supplementary file 2 — Table EV1 [file EMBJ-35-356-s002.docx]

Morrison_Table EV1

| Antibody | Dilution | Supplier |
| --- | --- | --- |
| Sox17 | 1:200 | R+D, AF1924 |
| Pdx1 | 1:200 | Chemicon, AB3505 |
| Ngn3 | 1:500 | Millipore, AB5684 |
| AFP | 1:100 | NeoMarkers, RB-365 |
| FoxA2 | 1:200 | Santa Cruz, sc-6554 |
| Ecadherin | 1:1000 | Takara Bio Inc, M107 |
| CXCR4/CD184 | 1:200 | BD Biosciences, 551852 |
| PDGFRa/CD140a | 1:200 | eBioscience, 17-1401 |
