# Supplementary figures and images for: Convergence of cMyc and β‐catenin on Tcf7l1 enables endoderm specification
Source: EMBO J. 2015 Dec 16;35(3):356–68. doi: 10.15252/embj.201592116 (PMC4741304; doi:10.15252/embj.201592116)

**Figure 2B**

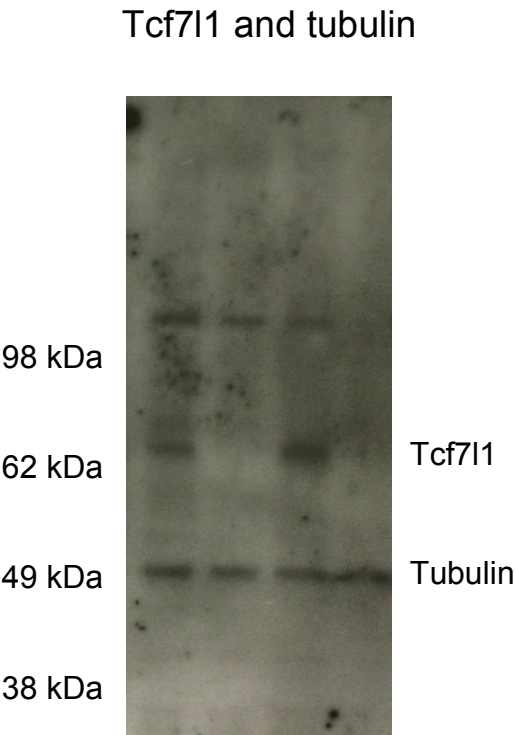

Tcf7l1 expected size 68.2 kDa  
Tubulin expected size 50 kDa

Supplement: Supplementary file 5 — Source Data for Figure 2 [file EMBJ-35-356-s004.pdf]

Figure 5D.

Tcf7l1

98 kDa  
62 kDa  
49 kDa  
38 kDa  
28 kDa  
17 kDa

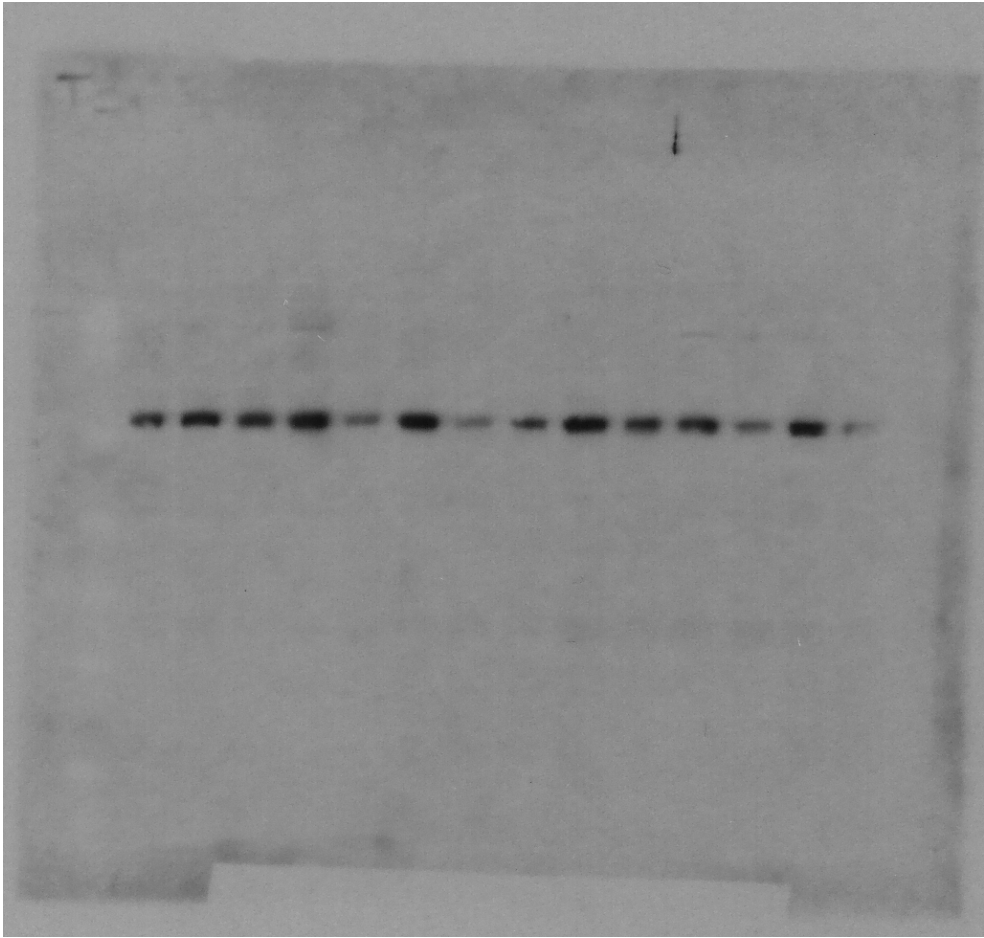

Tubulin

98 kDa  
62 kDa  
49 kDa  
38 kDa  
28 kDa  
17 kDa

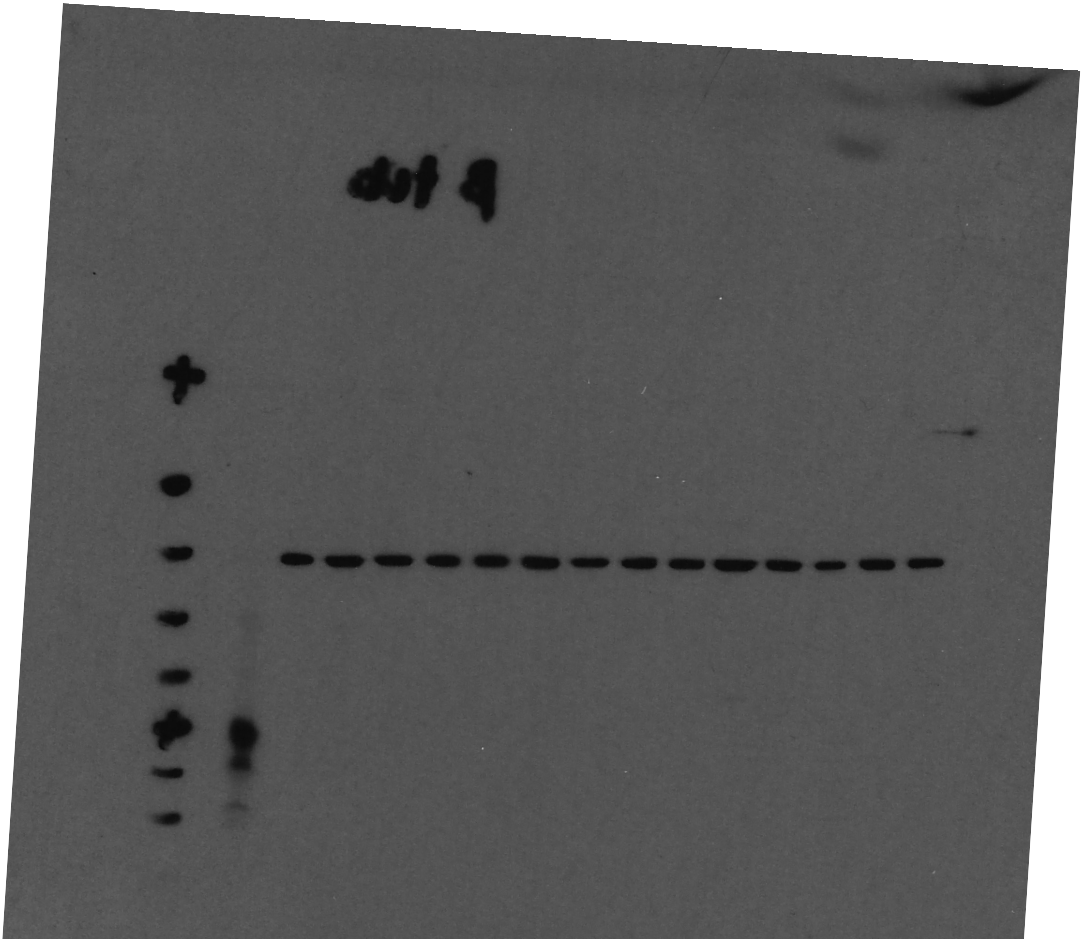

Supplement: Supplementary file 6 — Source Data for Figure 5 [file EMBJ-35-356-s005.pdf]
