# Supplementary material for: Convergence of cMyc and β‐catenin on Tcf7l1 enables endoderm specification
Source: EMBO J. 2015 Dec 16;35(3):356–68. doi: 10.15252/embj.201592116 (PMC4741304; doi:10.15252/embj.201592116)
Supplement: Supplementary file 7 — Source Data for Figure 6 [file EMBJ-35-356-s006.pdf]

Figure 6B.

Total cMyc

98 kDa
62 kDa
49 kDa
38 kDa
28 kDa
14 kDa

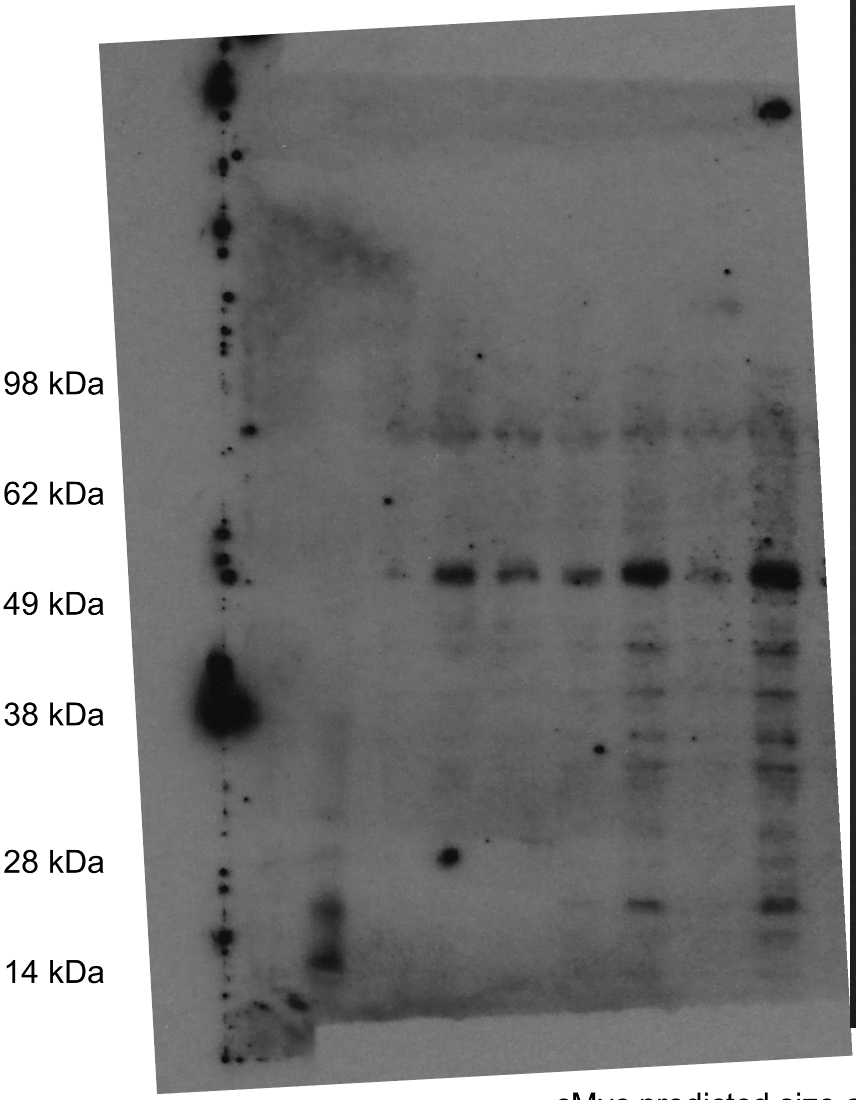

pSer62 cMyc

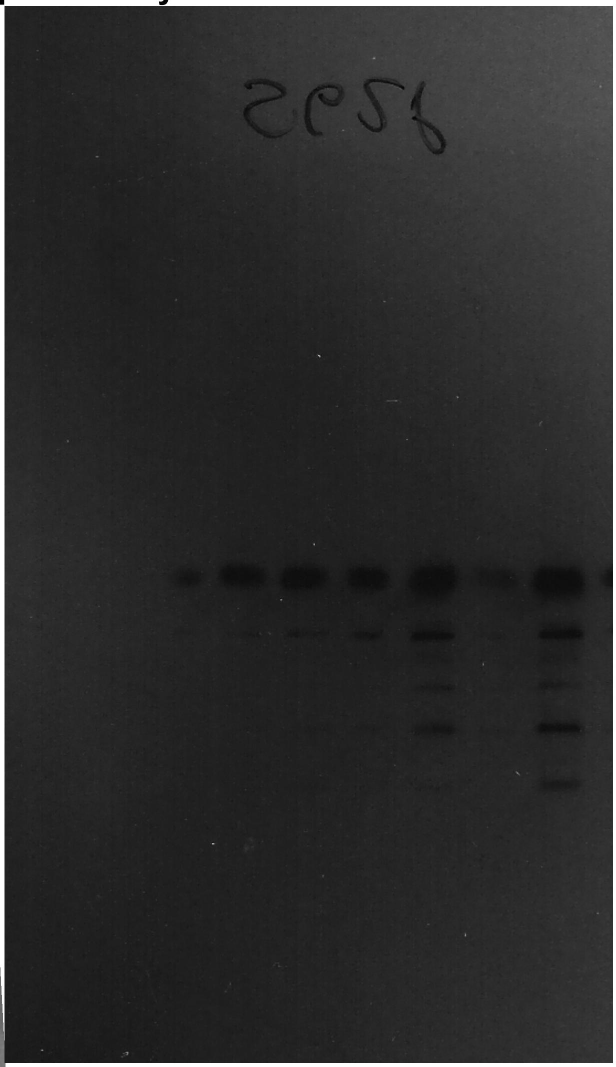

cMyc predicted size approx. 55 kDa

Tubulin

98 kDa
62 kDa
49 kDa
38 kDa
28 kDa

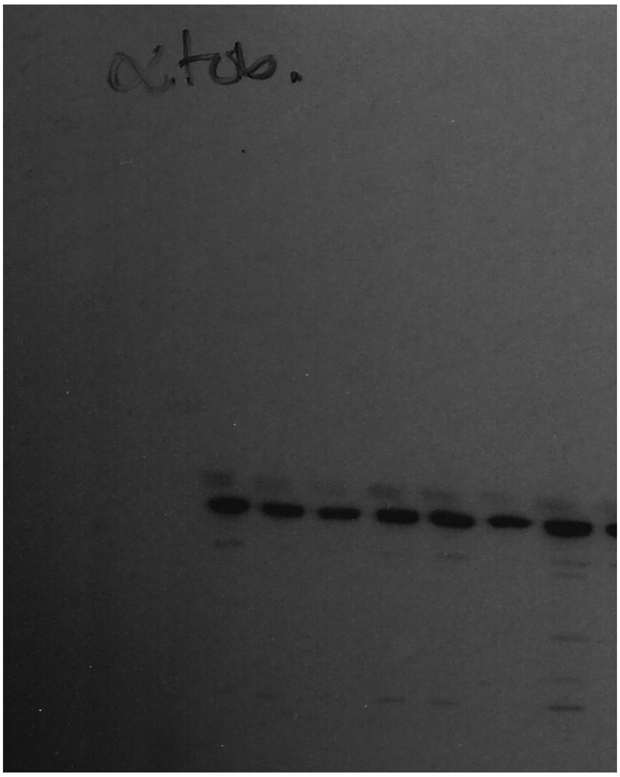

First lane for all 3 blots
= ES cells

[illegible]

Figure 6C.

pThr358 cMyc and cMyc

98 kDa
62 kDa
49 kDa
38 kDa
28 kDa
14 kDa

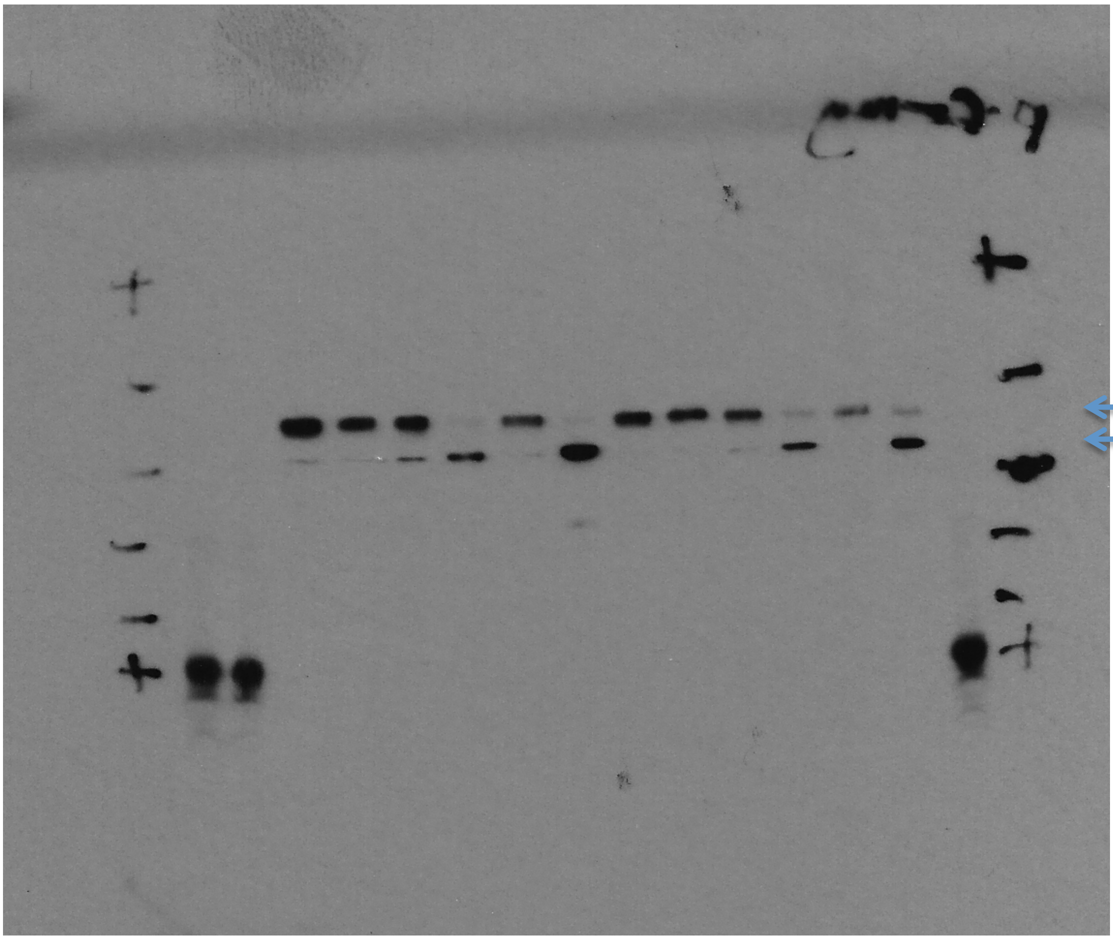

pThr358 cMyc
cMyc

cMyc predicted size 55 kDa,
pThr385cMyc upper band

Tubulin

98 kDa
62 kDa
49 kDa
38 kDa

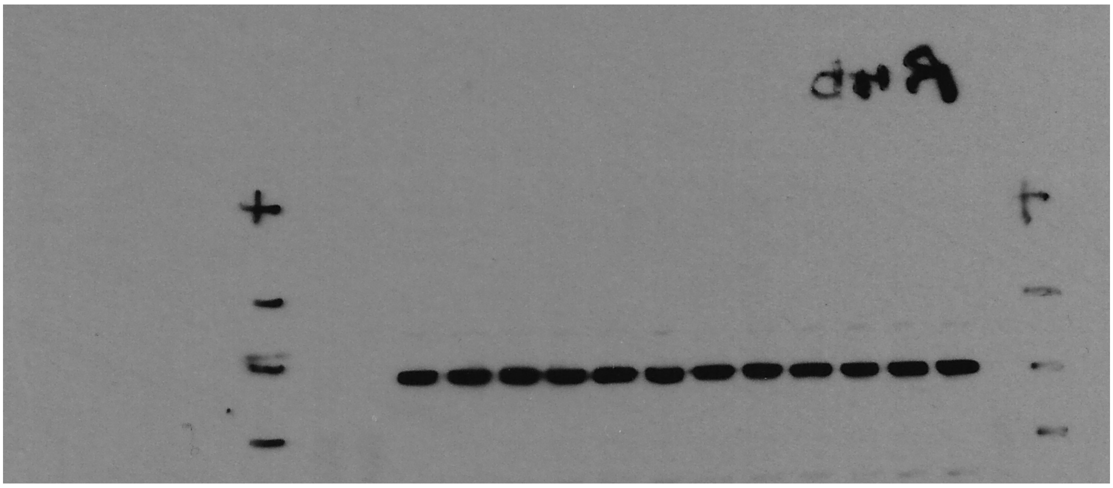

Figure 6D.

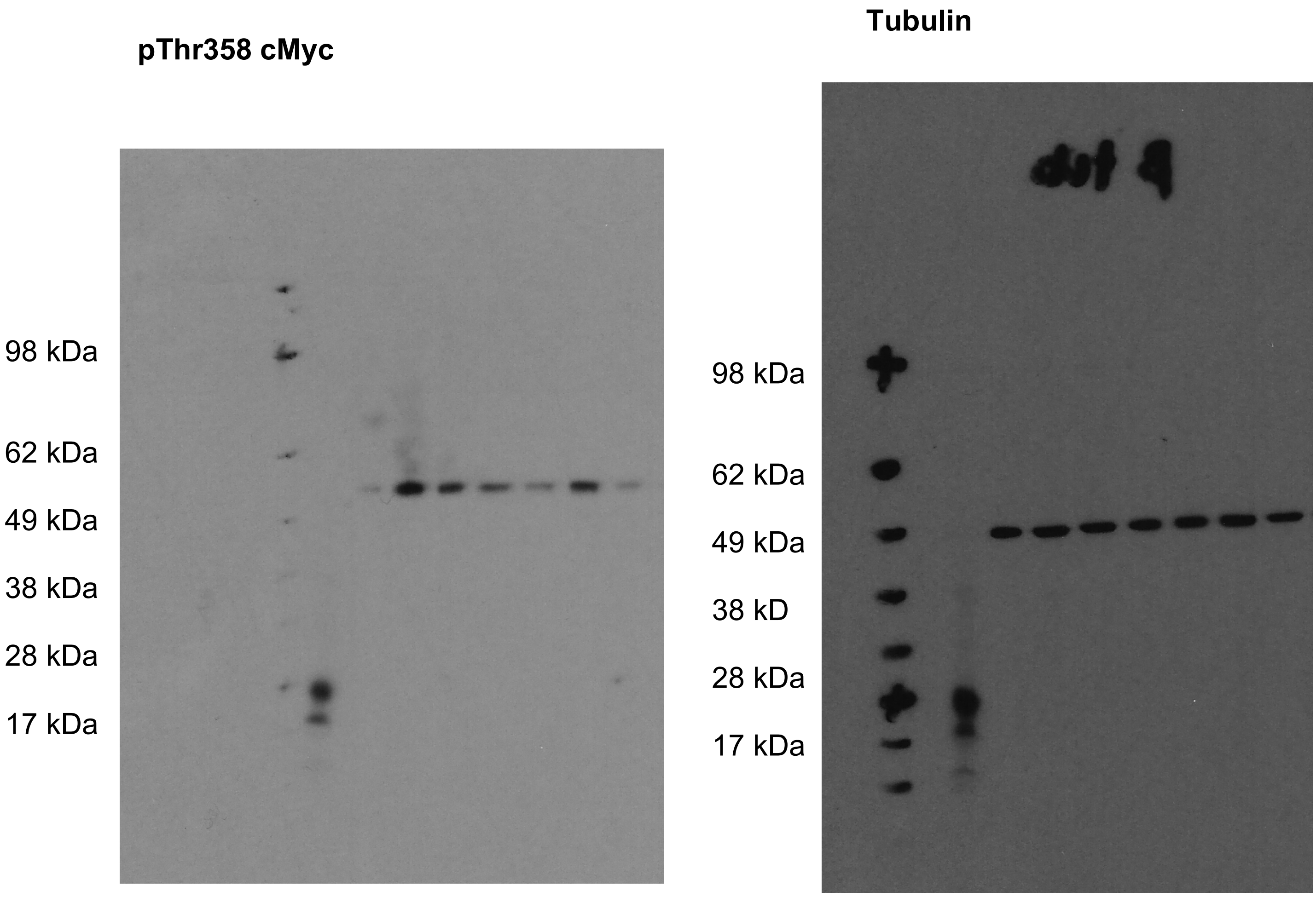

First lane = ES cells

Figure 6J.

Total cMyc

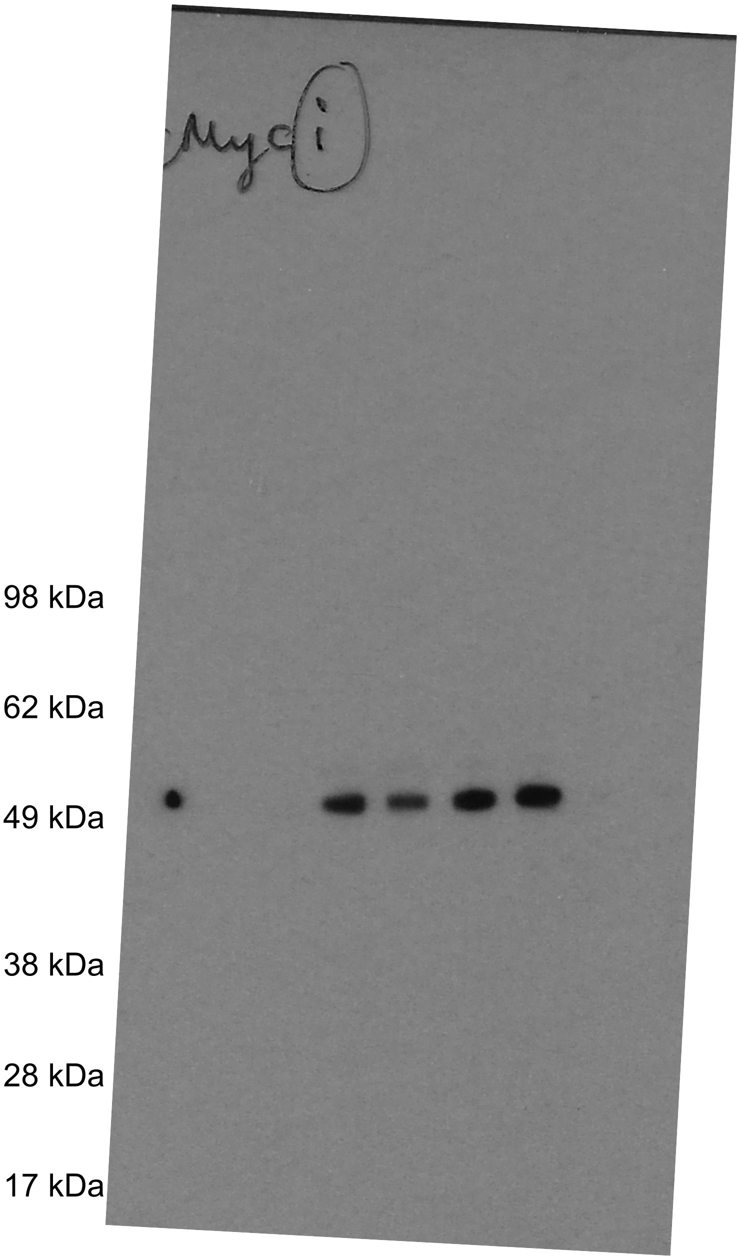

Tcf7l1

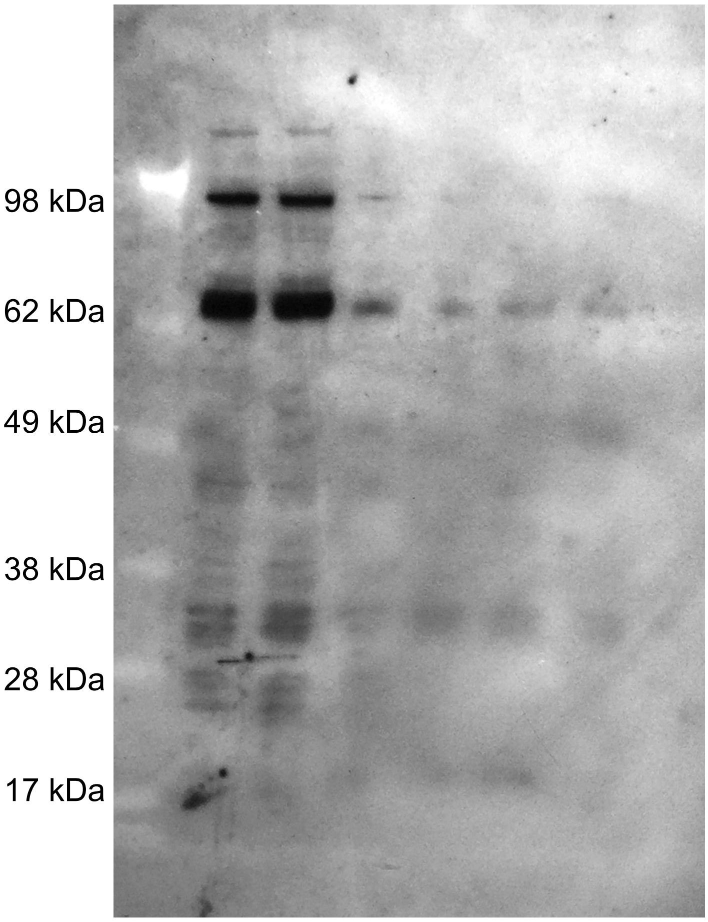

Tcf7l1 predicted size 68.2 kDa
